# Comparing Steady-State Visually Evoked Potentials Frequency Estimation Methods in Brain-Computer Interface With the Minimum Number of EEG Channels

**DOI:** 10.32598/bcn.9.10.200

**Published:** 2019-05-01

**Authors:** Mehrnoosh Neghabi, Hamid Reza Marateb, Amin Mahnam

**Affiliations:** 1. Department of Biomedical Engineering, Faculty of Engineering, University of Isfahan, Isfahan, Iran.

**Keywords:** Brain-Computer Interface (BCI), Electroencephalogram (EEG), Feature extraction, Steady-State Visually Evoked Potential (SSVEP)

## Abstract

**Introduction:**

Brain-Computer Interface (BCI) systems provide a communication pathway between users and systems. BCI systems based on Steady-State Visually Evoked Potentials (SSVEP) are widely used in recent decades. Different feature extraction methods have been introduced in the literature to estimate SSVEP responses to BCI applications.

**Methods:**

In this study, the new algorithms, including Canonical Correlation Analysis (CCA), Least Absolute Shrinkage and Selection Operator (LASSO), L1-regularized Multi-way CCA (L1-MCCA), Multi-set CCA (MsetCCA), Common Feature Analysis (CFA), and Multiple Logistic Regression (MLR) are compared using proper statistical methods to determine which one has better performance with the least number of EEG electrodes.

**Results:**

It was found that MLR, MsetCCA, and CFA algorithms provided the highest performances and significantly outperformed CCA, LASSO, and L1-MCCA algorithms when using 8 EEG channels. However, when using only 1 or 2 EEG channels d, CFA method provided the highest F-scores. This algorithm not only outperformed MLR and MsetCCA when applied on different electrode montages but also provided the fastest computation time on the test set.

**Conclusion:**

Although MLR method has already demonstrated to have higher performance in comparison with other frequency recognition algorithms, this study showed that in a practical SSVEP-based BCI system with 1 or 2 EEG channels and short-time windows, CFA method outperforms other algorithms. Therefore, it is proposed that CFA algorithm is a promising choice for the expansion of practical SSVEP-based BCI systems.

## Highlights

- The Common Feature Analysis (CFA) method provides the highest F-scores in comparison with Multivariate Linear Regression (MLR) and Multiset Canonical Correlation (MsetCCA) when only one or two EEG channels are used.
- The MLR method could use information in multichannel EEG towards providing higher F-scores.
- The CFA method was less sensitive to the placement of Oz electrode compared with the other methods.
- Oz-Pz montage has better F-score value than the other monopolar montages.
- The CFA algorithm takes the lowest computational time which is important for online implementation of the brain-computer interface system.

## Plain Language Summary

Brain-Computer Interface (BCI) is a new way of communication for people with disabilities to improve their quality of life. Applications include spelling for communication, control of artificial limbs, and home automation systems. Steady-State Visual-Evoked Potential (SSVEP)-based BCI systems are widely used in recent decades because of their high information transfer rate and easy training phase. Different methods have been introduced in the literature to estimate SSVEP responses for BCI applications. In this study, these new algorithms are compared using proper statistical methods to determine which one has better performance when a few numbers of EEG electrodes are used. This is important for developing a practical BCI system. Our results show that the Common Feature Analysis (CFA) method provides the highest accuracy in comparison with the other methods when only one or two EEG channels are used. Also, it is less sensitive to the placement of the electrodes compared with the other methods, and it has the lowest computational time (which is important for online implementation of a BCI system). Therefore, the CFA method is a promising choice for the expansion of practical SSVEP-based BCI systems.

## 1. Introduction

Brain-Computer Interface (BCI) can provide a new way of communication and control between the human brain and external devices. Although this communication method could be used by everyone, its applications have been mostly used for the patients with disabilities so far (Resalat & Saba, 2016) to improve their quality of life (Tello, Müller, Bastos-Filho, & Ferreira, 2014). Recently, many new BCI applications have been validated for disabled people, including neuroprostheses, spelling for communication, control of artificial hands and legs, and playing with games (Zhang, Xu, Cheng, & Yao, 2014a).

There are different techniques to detect brain activities for the realization of a BCI system, including Electroencephalography (EEG), functional near-infrared spectroscopy, Magnetoencephalography (MEG) and functional Magnetic Resonance Imaging (fMRI). Recording EEG signals from the scalp is a common approach with the neural data signals for BCI devices since it can be easily recorded in most of the circumstances by rather simple equipment. Also, it has a high temporal resolution (Zhang, Zhou, Jin, Wang, & Cichocki, 2014b).

A variety of EEG-based BCI paradigms have been proposed based on different brain responses to transfer the user intent to the computer, such as Sensorimotor Rhythms (SMRs), Slow Cortical Potentials (SCPs), P300 Event-Related Potentials (ERPs), and Steady-State Visually Evoked Potentials (SSVEP) (Zhang et al., 2014b). Recently research on SSVEP-based BCI systems has been extended because of their high Information Transfer Rate (ITR) and easy training phase (Ge et al., 2017).

SSVEP is a neurophysiological reaction excited in the occipital and occipitoparietal region of the brain by a flickering visual stimulus at a specified frequency. Responses of SSVEP contain the basic frequency of the visual stimulus and some of its harmonics are sometimes accompanied with a sub-harmonic. Therefore, SSVEP-based BCI systems understand the user commands when the user looks at one stimulus by identifying the corresponding frequency components in the EEG (Wang et al., 2016).

Different approaches have been introduced in the literature to estimate SSVEP responses for BCI applications (Liu, Chen, Ai, & Xie, 2014). A conventional method is the Power Spectral Density Analysis (PSDA) (Wei, Xiao, & Lu, 2011). However, when using this algorithm, the length of processing time window has to be typically more than 3 s to have acceptable frequency resolution, resulting in low ITR (Ming, Xiaorong, Shangkai, & Dingfeng, 2002).

Canonical Correlation Analysis (CCA) (Lin, Zhang, Wu, & Gao, 2007) is a method for exploring the relationships between two multivariate sets of vectors. One of these variables is the EEG signal from various channels and the other is the artificial sine-cosine signal. Researchers have demonstrated that CCA usually outperforms PSDA (Wang et al., 2014). Moreover, high accuracy BCI systems could be developed using CCA in which their required data frame is as short as 2 s (Zhang, Jin, Qing, Wang, & Wang, 2012).

Zhang et al. (2011) (Cited by Wang et al., 2016) proposed a multi-way development of standard CCA (MCCA) by inspecting the correlation among various variables, including space and trial modes of multidimensional EEG data and sine-cosine signals. MCCA and its L1-regularized development (L1-MCCA) (Zhang et al., 2013) have been introduced to supply refined SS-VEP recognition fulfillment in comparison with standard CCA (Wang et al., 2016).

Least Absolute Shrinkage and Selection Operator (LASSO) algorithm (Zhang et al., 2012) is another method proposed in the literature for computing the contribution of different stimulus frequencies and their harmonics in the recorded EEG signal. The frequency with maximum contribution degree was identified as the goal frequency. LASSO algorithm has been shown to improve frequency identification performance with a shorter time window compared with CCA algorithm (Liu et al., 2014).

Zhang et al. (2014b) introduced a Multi-set CCA method (MsetCCA) for frequency identification in SSVEP. In this method, training information is used for reference signals (Zhang et al., 2014b). Accuracy of MsetCCA approach is more than CCA and MCCA methods when using time windows shorter than 2 s (Zhang et al., 2014b).

Zhang et al. (2015) considered that a set of EEG signals would share certain common components in response to a specified stimulus on different subjects. These EEG signals contain common components which may have characteristics of SSVEP responses. Therefore, these components could be further efficient reference data for SSVEP identification in using correlation methods. So they proposed a Common Feature Analyzes (CFA) method (Zhang, Zhou, Jin, Wang, & Cichocki, 2015). It outperformed SSVEP identification accuracy compared with those of the CCA and the MCCA methods in a 0.5-s time window (Zhang et al., 2015).

Another approach that has been recently proposed for distinguishing features of SSVEP is Multivariate Linear Regression (MLR) (Wang et al., 2016). This algorithm outperformed CCA and MCCA algorithms and especially provided higher classification accuracies when using a short-time window of 1 s (Wang et al., 2016). The above-mentioned methods and their properties are listed in Table 1.

**Table 1.** Different methods used for SSVEP recognition in BCI

**Method**	**Concept**	**Training Requirement**	**Reference**	**The Number of EEG Channels Used in the Study**
PSDA	Significant peaks at the frequencies of the stimuli are detected from Power Spectral Density of the user’s EEG signal within a time window	_	(Ming et al., 2002)	2
CCA	A method for exploring the relationship between two multivariate sets of vectors	_	(Lin et al., 2007)	8
MCCA	It uses the optimal reference signals after adjustment, with increased computational time	Yes	(Yu Zhang et al., 2011b)	8
L1MCCA	This method is an extension of the CCA for reference signal optimization	Yes	(Yu Zhang et al., 2013)	8
LASSO	It assumes that SSVEPs are standard linear regression models of stimulation signals	_	(Yu Zhang et al., 2012)	3
MsetCCA	An extension of CCA to recognize multiple linear transforms to optimize signal references with EEG signals	Yes	(Yangsong Zhang et al., 2014)	8
CFA	A method to exploit the latent common features shared by a set of EEG signals experiments as the improvement reference	Yes	(Yu Zhang et al., 2015)	8
MLR	Multivariate Linear Regression is implemented to exploit the distinguished SSVEP components	Yes	(H. Wang et al., 2016)	8

With the advancement in BCI research, these systems are coming out of the lab for practical applications. In addition to high accuracy and information transfer rate, user comfort is critical. It must be met at least in a level that a potential user accepts and continues to work with the system (Qing, Zheng, Yue, Yuankui, & Ge, 2015).

One of the significant factors to ensure user comfort is that the BCI system works with only a few EEG electrodes. A large number of electrodes increases the preparation time beyond the acceptable level for practical use and increases the size and final cost of the system. On the other hand, achieving high and or enough accuracy and information transfer rate with a minimum number of electrodes is challenging due to the reduction of their available information. As shown in Table 1, most of the studies that proposed and compared algorithms for SS-VEP detection used a large number of EEG channels.

The goal of this study was to ascertain which of the newly-proposed algorithms (reviewed above) is more appropriate for the development of a practical SSVEP-based BCI system with high accuracy and information transfer rate when using a minimum number of electrodes. Here CCA (Lin et al., 2007), LASSO (Zhang et al., 2012), L1MCCA (Zhang et al., 2013), MsetCCA (Zhang et al., 2014b), CFA (Zhang et al., 2015) and MLR (Wang et al., 2016) methods are compared using proper statistical methods to determine their performance in real applications.

The rest of the paper is organized as follows. In the next section, information about the experimental protocol and the BCI methods used in this study as well as the statistical methods are presented. Section 3 supplies the results of the performance assessment. Ultimately, the discussion is provided in Section 4.

## 2. Methods

Numerous methods have been proposed for SSVEP frequency recognition in the literature. Of those, LASSO (Zhang et al., 2012), L1-MCCA (Zhang et al., 2013), MsetCCA (Zhang et al., 2014b), CFA (Zhang et al., 2015), and MLR (Wang et al., 2016) have demonstrated better performances and therefore we selected them for this study. Also, CCA method is a benchmark method and thus we selected it for this study. These algorithms are described as follows: SSVEP detection based on CCA; SSVEP detection based on LASSO; SSVEP detection based on L1MCCA; SSVEP detection based on MsetCCA; SSVEP detection based on CFA; SSVEP detection based on MLR.

CCA is a multivariate statistical method to explore the underlying correlation between two sets of data. The first set (X) is the EEG recorded from several channels while the second one (Y) is the sine-cosine reference signals reconstructed as below:

(1) $$Y=\left[\begin{array}{c} \sin(2\pi (f_{i})t)\\ \cos(2\pi (f_{i})t)\\ \vdots \\ \sin(2\pi (Nf_{i})t)\\ \cos(2\pi (Nf_{i})t) \end{array}\right];t=\frac{1}{F_{\text{S}}},\frac{2}{F_{\text{S}}},\ldots ,\frac{n}{F_{\text{S}}}$$,

where N refers to the number of harmonics, f_i_ denotes the i^th^ stimulus frequency (fundamental frequency), n represents the number of sampling points, and F_s_ is the sampling rate.

CCA tries to seek a pair of linear transforms, W_x_ and W_y_ that maximizes the correlation between x=X^T^W_x_ and y=Y^T^W_y_. The following optimization problem is solved for each frequency (Zhang et al., 2011).

(2) $$p_{i}=\underset{W_{x}W_{y}}{\max}\frac{E[x^{T}y]}{\sqrt{E[x^{T}x]E[y^{T}y]}}=\frac{W_{x}^{T}XY^{T}W_{y}}{\sqrt{W_{x}^{T}XX^{T}W_{x}Y^{T}W_{y}Y^{T}W_{y}}}$$,

where p_i_ is the association between the recorded signals and the synthetic waveform of the i^th^ frequency. The frequency with the maximum correlation coefficient is selected as the target frequency (Lin et al., 2007).

In LASSO algorithm, each EEG trial assumes that SSVEPs are standard linear regression models ( Eq. 1) for the response y ∈ R^n^.

(3) y=Xβ+ε,

where X=(x_1_,x_2_,...,x_p_) denotes the predictor variables, y is the response, and *ε* represents a noise vector. LASSO approximation is given by:

(4) $$\hat{\beta }=\underset{\beta }{\mathrm{argmin}}({{\left\|y-X\beta \right\|}^{2}}_{2}+\lambda {\left\|\beta \right\|}_{1})$$,

where, ‖•‖_1_, ‖•‖_2_ demonstrate the l_1_-norm and l_2_-norm, respectively (Tibshirani, 2011) and λ is a penalty parameter. The optimization problem demonstrated by Eq. (2) was resolved by quadratic programming (Schittkowski, 1986).

To create the model of SSVEP identification, a symmetric square-wave signal X, corresponding to the stimulus frequencies, is considered as the reference signal shown in Eq. (3).

(5) $$X=\left[\begin{array}{c} \sin(2\pi (f_{i})t)\\ \cos(2\pi (f_{i})t)\\ \vdots \\ \sin(2\pi (Nf_{i})t)\\ \cos(2\pi (Nf_{i})t) \end{array}\right];t=\frac{1}{F_{\text{S}}},\frac{2}{F_{\text{S}}},\ldots ,\frac{n}{F_{\text{S}}}$$,

where N refers to the number of harmonics, f_i_ denotes the i^th^ stimulus frequency (fundamental frequency), n represents the number of sampling points, and F_S_ is the sampling rate.

The LASSO estimator β̂ among the EEG signals y and the artificial reference set X were calculated by Eq. (2).

Then the contribution degree of each stimulus frequency is calculated for all the recorded channels of EEG as:

(6) $${\mathrm{CD}}_{i}=\frac{{\sum^{M}}_{k=1}{\sum^{2K}}_{j=1}\left|{\beta^{k}}_{i,j}\right|}{M}$$,

where M equals the number of channels, K denotes the number of harmonics, and CD_i_ refers to the contribution degree of the i_th_ square-wave in the signal. The maximum contribution implies the target frequency which the subject gazing at (Zhang et al., 2012).

L1-regularization (L1-MCCA) was proposed in the literature to give a function which can automatically select features for optimizing reference data in SSVEP-BCI detection (Zhang et al., 2017).

To construct the SSVEP recognition model, consider a three-way tensor X ∈ R^I×n×K^ (channel×time×experiment) formed by EEG signals recorded from some channels out of numerous experiments with a particular stimulus frequency and a signal collection Y ∈ R^2N×n^ shown in Eq. (3).

The optimization problem in L1-MCCA is formulated as:

w1,w3,v=argminw1,w3,v12‖X×w1T1×w3T3−vTY‖22+λ1‖w1‖1+λ2‖v‖1+λ3‖w3‖1

(7) $$s.t.\;\;{\left\|w_{1}\right\|}_{2}={\left\|w_{3}\right\|}_{2}={\left\|v\right\|}_{2}=1$$,

where w_1_ ∈ R^I^, w_3_ ∈ R^K^, v ∈ R^2N^ are projection vectors and λ_1_, λ_2_. λ_3_ are adjustment parameters. The LASSO estimation is equivalent to (5) (Tibshirani, 2011; Zhang et al., 2012) when any two of w_1_, w_3_, and v are constant. This problem would be resolved by an alternating LASSO method (Zhang et al., 2013).

The Multiset CCA (MsetCCA) method was recently proposed for reference data optimization from a common component in numerous calibration experiments (Nakanishi, Wang, Wang, & Jung, 2015). MsetCCA was extended for correlation maximization among canonical variables from numerous collections of random variates with distinguishing multiple linear transforms (Zhang et al., 2014b). Assume numerous groups of random variables X_i_ ∈ R^I_i_×J^ (i=1,2,…,N). In order to maximize the throughout correlation across canonical variables, the MAXVAR objective function is characterized as:

(8) $$\begin{array}{l} \underset{w_{1},\ldots ,w_{N}}{\max}\rho ={\sum^{N}}_{i\neq j}w_{i}^{T}C_{ij}w_{j}\\ s.t.\frac{1}{N}{\sum^{N}}_{i=1}{w^{T}}_{i}C_{ii}w_{i}=1, \end{array}$$,

where C_ij_ =X_i_X_j_^T^ is the between-set covariance matrix, and ρ is the correlation coefficient. The objective function in (6), may be transformed into the eigenvalue problem with the approach of Lagrange multipliers as:

(9) (R−S)w=ρSw,

where

$$\begin{array}{l} R=\left[\begin{array}{ccc} C_{11} & \cdots & C_{1N}\\ \vdots & \ddots & \vdots \\ C_{N1} & \cdots & C_{NN} \end{array}\right],\\ S=\left[\begin{array}{ccc} C_{11} & \cdots & 0\\ \vdots & \ddots & \vdots \\ 0 & \cdots & C_{NN} \end{array}\right],\;\;\;\;\;\;W=\left[\begin{array}{c} W_{1}\\ \vdots \\ W_{N} \end{array}\right]. \end{array}$$

Assume, X_1,m_, X_2,m_, ..., X_N,m_ ∈ R^C×P^ (C channels×P points) demonstrate EEG signals groups, including experiments at the m-th stimulus frequency f_m_. The MsetCCA is performed to distinguish numerous spatial filters w_1,m_, w_2,m_, ..., w_N,m_, to maximize throughout correlation between the canonical variables z̃_1,m_, z̃_2,m_, ...,z̃_Nm_ with the linked spatial filtering z̃_i,m_ =w^T^_i,m_X_i,m_ (i=1,2,…,N). These canonical variables show the common components between numerous training signals considered to have better accuracy of the real SSVEP specifications in comparison with sine-cosine reference signals. Canonical variables were combined to construct the reference signal optimization at frequency f_m_. These optimized reference signals are defined as:

(10) $$Y_{m}={[{{\tilde{z}}_{1,m}}^{T},{{\tilde{z}}_{2,m}}^{T},\ldots ,{{\tilde{z}}_{N,m}}^{T}]}^{T}.$$

For each stimulus frequency f_m_, the corresponding reference signal Y_m_ is consumed for computing the maximum correlation coefficients with the EEG signals (Zhang et al., 2014b).

Another approach is to explicitly model the common and distinct components. This approach has a similar concept with the multiset approach (Wang et al., 2016) to exploit the common features which multiple EEG signals share at the same stimulus frequency.

In this method, a set of matrices X={X ∈X^I_k_×T^:k=1,2,…,K}, share at least one common dimension T. They can be, for example, a set of multichannel EEG signals (channel×time×point) recorded for the same visual stimulus but from different subjects. These data matrices can be factorized in a linked way shown in Eq. (9):

(11) $$\begin{array}{l} X_{k}=A_{k}{B^{T}}_{k}=[{\bar{A}}_{k}{\overset{ˇ}{A}}_{k}]\left[\begin{array}{c} {\bar{B}}^{T}\\ {{\overset{ˇ}{B}}^{T}}_{k} \end{array}\right]={\bar{A}}_{k}{\bar{B}}^{T}+{\overset{ˇ}{A}}_{k}{{\overset{ˇ}{B}}^{T}}_{k}={\bar{X}}_{k}+{\overset{ˇ}{X}}_{k}\\ \forall \;\;k=1,2,\ldots ,K \end{array}$$,

where B̄ ∈R^T×C^, B̌_k_ ∈R^T×R̃^_k_, R_k_ =R̃_k_ +C is the number of latent components with R_k_<I_k_ and C is the number of common components. Finally, Ā_k_ and Ă_k_ are the partitions of the mixing coefficients A corresponding to B̄ and B̌_k_. For M stimulus frequencies, the common features B̄_k_(k=1,2,..., K) at each frequency would be exploited for a new test signal x̌∈X^RT^ ; the target frequency is identified as (Zhang et al., 2015):

(12) $$f_{t}=\underset{fk}{\mathrm{argmax}}{\left\|{\hat{x}}^{T}B_{k}\right\|}_{2},\;\;\;(k=1,2,\ldots ,K)$$

MLR is a technique for modeling the relationship between a scalar dependent vector and one or more independent vectors. Consider EEG training data X=[x_1_,x_2_,…,x_N_]∈R^D×N^, where D demonstrates the dimension of feature (D=C channels×P temporal points) and N is the number of signal points. Corresponding to the training points, a matrix of the label is created, using one of M class coding. The goal of MLR is to find distinguished subspaces by minimizing the objective function as follows:

(13) $$\begin{array}{l} \underset{W,b}{\min}\frac{1}{2}{{\left\|(y^{\left(i\right)}-(W^{T}{\tilde{x}}^{\left(i\right)}+b)\right\|}^{2}}_{2}\\ W,b \end{array}$$,

where W=[w_1_,w_2_,…,w_C_]∈R^S×C^ demonstrates the projection matrix and b is the model separation.

The model of MLR is then considered as:

(14) $$W=\underset{W}{\mathrm{argmin}}\frac{1}{2}{\sum^{N}}_{i=1}{{\left\|y^{\left(i\right)}-(W^{T}{\tilde{x}}^{\left(i\right)})\right\|}^{2}}_{2}$$

or in the vectorized form:

(15) $$W=\underset{W}{\mathrm{argmin}}\frac{1}{2}{{\left\|Y-W^{T}\tilde{X}\right\|}^{2}}_{F}$$,

where ‖.‖_F_ demonstrates the Frobenius norm. Then, the optimal solution is provided by:

(16) $$W={(\tilde{X}{\tilde{X}}^{T})}^{†}\tilde{X}Y^{T}$$,

where (.)^†^ demonstrates the Moore-Penrose pseudoinverse. The columns of W represent the features of training data. Ultimately, the k-NN (5-NN) classifier is used to classify the sub-space features exploited via the MLR (Wang et al., 2016).

### 2.1. Data Description

EEG data from an online available dataset (Wang et al., 2016) were used in our study. The dataset contained data from ten subjects (all males, aged from 21 to 27 years) and recorded from 8 channels (P7, P3, Pz, P4, P8, O1, Oz and O2). The sampling rate was fixed at 250 Hz. During the experiment, the participants were seated in a comfortable armchair 60 cm away from the center of the monitor. The experiment was performed in a shielded room. The EEG data were then bandpass filtered from 4 to 45 Hz. Four frequencies (6, 8, 9, and 10 Hz) were adopted in a recording session. The subjects were asked to gaze at each stimulus frequency for 4 s. Overall, there are 80 trials in this dataset. This dataset has been widely used in the literature as the benchmark (Wang et al., 2016; Zhang et al., 2012; Zhang et al., 2014b; Zhang et al., 2013; Zhang et al., 2015).

### 2.2. Experimental evaluation

In this study, the new algorithms, including MLR and CFA are compared with MsetCCA, L1MCCA, LASSO, and CCA algorithms to assess their efficacy for target frequency detection in a practical BCI system. The number of harmonics needed to define reference signals was set to 2 for CCA, LASSO, and L1-MCCA algorithms.

In LASSO and L1-MCCA methods, the lambda parameters were set to 0.5 and 0.02, respectively (Zhang et al., 2012; Zhang et al., 2013). The leave-one-run-out cross-validation was used to assess the average detection accuracy in the entire analyzed methods. The signals from 19 runs were used as training signals whereas the signals from the left-out runs were used for validation (Zhang et al., 2014b).

### 2.3. Validation

The performance of the methods was assessed using the F-score (F1 score). It was shown in the literature that this criterion is more suitable than the overall accuracy of multi-class problems (Sokolova & Lapalme, 2009) because the latter index overestimates the performance of the analyzed methods. Moreover, by using F-score, it is possible to analyze the performance of the system for each class better than the traditional accuracy measurements. For the calculation of F-score, first, the multi-class confusion matrix of four frequencies is created. Then, precision, sensitivity, and F-score are calculated for each frequency class as below:

(15) $$\mathrm{Pr}=\frac{\mathrm{TP}}{\mathrm{TP}+\mathrm{FP}}$$,

where Pr is the precision TP and FP and are the number of true and false positive predictions for each class.

(16) $$\mathrm{Se}=\frac{\mathrm{TP}}{\mathrm{TP}+\mathrm{FN}}$$,

where Se is the sensitivity and FN is the number of false negative predictions for each class.

(17) $$\mathrm{F-score}=\frac{2\times \mathrm{Pr}\times \mathrm{Se}}{\mathrm{Pr}+\mathrm{Se}}$$,

where F-score is the harmonic mean of the precision and sensitivity (=recall) of the corresponding class. This parameter is not dependent on TN (True Negative), and thus the performance of the analyzed system is not overestimated (Marateb, Mansourian, Adibi, & Farina, 2014).

### 2.4. Statistical methods

It is important to use proper statistical tests for rigorous comparison between different approaches, otherwise random or insignificant differences are considered as significant (Bossuyt et al., 2015; Karimimehr et al., 2017). The McNemar’s test (Webb & Copsey, 2011) was used to identify SSVEP frequency estimation algorithms outperforming the other methods. Pairwise comparisons were performed using the McNemar’s test. Then, the methods with higher and significant performance were shown as significantly outperforming methods.

For further analysis, GEE (Generalized Estimating Equation) (Hardin & Hilbe, 2007) was applied to determine any significant differences among the selected algorithms when different electrode montages (monopolar or bipolar) and the number of channels (1 or 2 channels) were used with repeated measurements in the time windows of 1 second. The level of statistical significance was considered 0.1 to be on the safe side and not to miss significant differences in this small sample size data (Lavrakas, 2008).

## 3. Results

**Figure 1.**
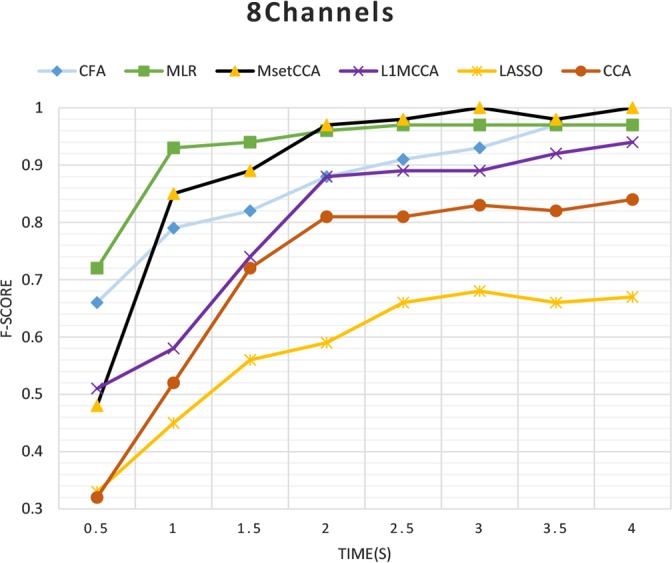
F-score of various methods for 8-channel analysis

We compared the performance of different SSVEP frequency estimation algorithms. Figure 1 demonstrates the resulting F-scores for time windows of 0.5 to 4 seconds when all 8 channels of recorded EEG were used. MLR method outperformed the other 6 methods in terms of mean F-score performance for the time windows of 0.5 to 1.5 s. MsetCCA and CFA methods were ranked second, while the performance of the CFA method was significantly higher than that of MsetCCA method for the time window of 0.5 s (P<0.05). Overall, LASSO had the lowest performance compared with the others.

**Figure 2.**
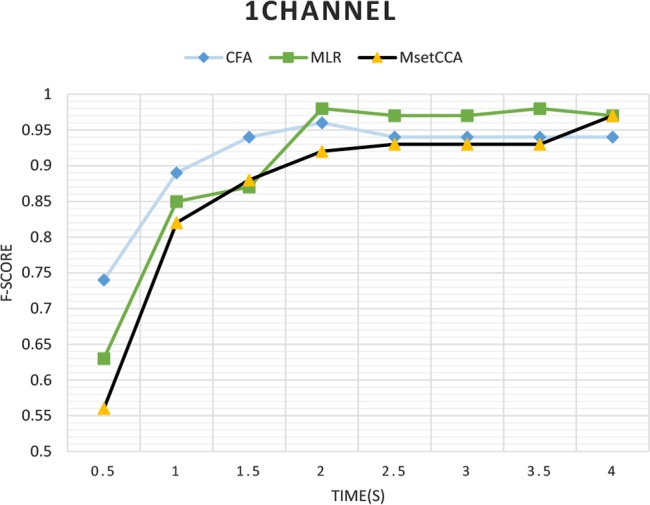
F-score of various methods for one-channel analysis

Overall, pairwise McNemar’s test of these results demonstrated that MLR, MsetCCA, and CFA algorithms significantly outperformed the other analyzed methods in the detection of gazed frequencies using 8 EEG channels (P<0.05). In fact, in the entire analysis windows, MLR, MsetCCA, CFA, L1-MCCA, CCA, and LASSO significantly outperformed 5, 3, 3, 2, 1, and 0 times in pairwise comparisons. Thus, only those three methods were studied in the rest of the paper. In a practical SSVEP-based BCI system, when only one channel of EEG is used, the performance of the algorithms might change compared with when using more channels. Therefore, the F-score measures of MLR, MsetCCA, and CFA algorithms were compared when only one channel of EEG (Oz-Pz channel) is used (Diez, Mut, Laciar, & Avila, 2010) (Figure 2).

For one-channel EEG, McNemar’s test was implemented to determine significant differences between CFA, MLR, and MsetCCA methods in different time windows (Table 2). In time windows smaller than 2 s, the CFA method had better F-score in target frequency recognition than MLR and MsetCCA methods. To do further extensive analysis, the F-score values for MLR, CFA, and MsetCCA methods were calculated for a time window of 1 s, when different electrode montages (monopolar or bipolar) and the number of channels (1 or 2 channels) were used (Table 2).

**Table 2.** Statistical analysis of F-score using McNemar’s test

**Time, s**	**0.5**	**1**	**1.5**	**2**	**2.5**	**3**	**3.5**	**4**	**0.5–4**

**Methods**									
CFA vs. MLR	NS	NS	NS	NS	NS	NS	NS	NS	NS
CFA vs. MsetCCA	CFA	NS	NS	NS	NS	NS	NS	NS	CFA
CFA vs. CCA	CFA	CFA	CFA	CFA	CFA	NS	NS	NS	CFA
MLR vs. MsetCCA	NS	NS	NS	NS	NS	NS	NS	NS	MLR
MLR vs. CCA	NS	NS	NS	MLR	MLR	MLR	MLR	MLR	MLR
MsetCCA vs. CCA	NS	NS	NS	NS	NS	NS	NS	MsetCCA	MsetCCA

NS: There is not a significant difference

The results are based on leave-one-out cross-validation test on all 20 trials of the data. CFA and MLR methods outperformed MsetCCA in the entire scenarios. Moreover, on average, CFA outperformed MLR method. Generalized Estimating Equation (GEE) test was applied to determine any significant differences between these algorithms when different scenarios (Table 3) were used. The result showed that CFA method significantly outperformed the other two algorithms (P<0.07). Also, MLR significantly outperformed MsetCCA method (P=0.001).

**Table 3.** F-score with different electrode montages and the number of channels

**Number**	**Channel**	**MsetCCA**	**CFA**	**MLR**
1	Oz-Pz, O1-P7	0.82	**0.97**	0.82
2	Oz-Pz, O1-P8	0.73	0.88	**0.9**
3	Oz-Pz, O2-P8	0.88	**0.97**	0.93
4	Oz-Pz, O2-P7	0.76	**0.98**	0.88
5	Oz-Pz, O1-Pz	0.84	0.85	**0.92**
6	Oz-Pz, O2-Pz	0.79	0.85	**0.89**
7	Oz-Pz, O1-Oz	0.84	**0.97**	0.92
8	Oz-Pz	0.81	**0.92**	0.84
9	O1-Pz	0.35	0.65	**0.69**
10	O2-Pz	0.45	**0.67**	0.58
11	O1, Oz	0.77	0.84	**0.86**
12	O2, Oz	0.82	0.85	**0.88**
13	O1, O2	0.4	**0.81**	0.78
14	Oz	0.78	**0.88**	0.86
15	O1	0.55	**0.76**	0.66
16	O2	0.56	**0.76**	0.66
Maximum		0.88	**0.98**	0.93
SD		0.173847	0.102337	0.109953
Mean		0.696875	0.850625	0.816875

The best result for the testing phase is displayed in bold.

The other important factor in the development of an online BCI system is the computational cost of the algorithms. The average running time for 19 runs of algorithms was obtained on a laptop with Windows 8.1 Operating System and 2.6 GHz Intel Core i5 CPU with 2 GB RAM and all of the algorithms were implemented in MATLAB 2014a (Table 4). It should also be stated that the MLR and CFA methods were implemented in the vectorized form.

**Table 4.** The average computational time (s) of various methods

**Time Window**	**Method**		**1 Channel**			**Methods**		

			**LASSO**	**L1-MCCA**	**CCA**	**MsetCCA**	**CFA**	**MLR**
1 s	Train	Mean	--	1.1881	--	0.0296	0.0338	0.0063
		SD	--	0.46853	--	0.00232	0.00036	0.00071
	Test	Mean	0.0082	0.0024	0.0045	0.0037	**0.0018**	0.0026
		SD	0.00115	0.00068	0.00136	0.00073	0.00064	0.00049
4 s	Train	Mean	--	1.5056	--	0.0315	0.0363	0.0097
		SD	--	0.44638	--	0.00256	0.00036	0.00114
	Test	Mean	0.0104	0.0028	0.0048	0.0055	**0.0019**	0.0027
		SD	0.00096	0.00041	0.00131	0.00050	0.00041	0.00040

The best result for the testing phase is displayed in bold.

In the training phase, MLR is the fastest algorithm and L1-MCCA is the most time-consuming algorithm. However, in applying the tuned algorithm to frequency detection in the test set, CFA provided the fastest computations, while LASSO took the longest computational time.

## 4. Discussion

In a practical SSVEP-based BCI system, it is important to use accurate frequency detection algorithms when a short-time window with only a few EEG channels are used for analysis. In this study, the state-of-the-art algorithms for SSVEP detection, including CCA, LASSO, L1-MCCA, MsetCCA, CFA, and MLR were compared on a benchmark database. The results demonstrated higher F-scores for MsetCCA, CFA and MLR methods in comparison with CCA, LASOO, and L1-MCCA methods for entire time windows when 8 channels of EEG are used. These results are in accordance with the results reported by Wang et al. (2016), demonstrating a higher performance for MLR method in comparison with CFA, MCCA, and CCA methods. Zhang et al. (2014b) suggested that MsetCCA provided a higher accuracy in comparison with MCCA and CCA methods.

Here it is demonstrated that MsetCCA also outperforms L1-MCCA. In another study, Zhang et al. (2015) showed that the CFA method provided higher accuracies than MCCA and CCA methods. Here it is demonstrated that CFA method also provides higher F-scores in comparison with L1-MCCA.

Further analysis demonstrated that while MLR method outperforms the other two algorithms when several EEG channels are used for analysis, it is the CFA method that provides the highest F-scores when only 1 or 2 EEG channels are used. We also compared the performance of CCA with a set of monopolar signals such as Oz and Pz or bipolar (Oz-Pz) signal with that of the other methods in Figure 2 (namely as CFA, MLR, MsetCCA). The results did not change and CFA and MLR methods still outperformed the other two algorithms for time windows shorter than 1 s.

The results presented in Table 3 can also be examined by another aspect. The 8th row in Table 3 represents the F-scores for Oz-Pz EEG channel, while in rows 1 to 7, the F-scores are provided when an additional bipolar channel was used. In most cases, the MLR method could effectively use additional information provided by the extra channels to improve the F-score of the system. In the CFA method, although the F-scores are higher, using an extra EEG channel does not have a consistently positive effect on the F-scores and the same can be observed for MsetCCA method. This capability of the MLR technique can also be observed when comparing the F-scores for Oz montage with Oz, O1 and Oz, O_2_ montages. Therefore, the MLR method could use information in multichannel EEG towards providing higher F-scores.

In practical applications of an SSVEP-based BCI system without the help of experts, the proper positioning of the electrodes is also a challenging issue. Therefore, another question is how much the algorithms should be robust to correct the position of electrodes. The results provided in Table 3 demonstrated that the performance of frequency detection algorithms is quite sensitive to the placement of Oz electrode. In fact, changing the position of this electrode to O1 or O2 considerably degrades the F-score of the system.

This result can be observed when Oz or Oz-Pz montages were used. However, the results show that the CFA method was less sensitive to the placement of Oz electrode compared with other methods. The comparison of F-score values between monopolar and bipolar montages in Table 3 indicates that Oz-Pz montage has better F-score value than other monopolar montages. This result is in accordance with the result reported by Diez et al. (2010).

The CFA algorithm also has the lowest computational time which is important for online implementation of a BCI system. Therefore, it is proposed that the CFA algorithm may be a proper choice in the development of practical SSVEP-based BCI systems. In the literature, different methods were only compared with CCA and MCCA methods (Zhang et al., 2012; Zhang et al., 2014b; Zhang et al., 2013; Zhang et al., 2015). Our study suggests that newly-developed algorithms could also be compared with CFA due to its higher performance for short-time windows when few EEG channels are used.

The limitations of the study are the small dataset, a small number of participants, and the limited number of frequencies. Therefore it is suggested to test these methods on a larger dataset. Moreover, more detailed analysis of sensitivity on the electrode placement can be performed when a large number of electrodes are used.
